# The Development of a Flexible Humidity Sensor Using MWCNT/PVA Thin Films

**DOI:** 10.3390/nano14201653

**Published:** 2024-10-15

**Authors:** Ana R. Santos, Júlio C. Viana

**Affiliations:** IPC—Institute for Polymers and Composites, University of Minho, 4804-533 Guimarães, Portugal; id10233@alunos.uminho.pt

**Keywords:** carbon nanotubes, polyvinyl alcohol, humidity, flexible, sensor

## Abstract

The exponential demand for real-time monitoring applications has altered the course of sensor development, from sensor electronics miniaturization, e.g., resorting to printing techniques, to low-cost, flexible and functional wearable materials. Humidity sensing has been used in the prevention and diagnosis of medical conditions, as well as in the assessment of physical comfort. This paper presents a resistive flexible humidity sensor composed of silver interdigitated electrodes (IDTs) screen printed onto polyimide film and an active layer of multiwall carbon nanotubes (MWCNT) dispersed in a water-soluble polymer, polyvinyl alcohol (PVA). Different MWCNT/PVA sensor sizes and MWCNT percentages are tested to study their effect on the initial electrical resistance (R_i_) values and sensor response at different humidity percentages. The results show that the R_i_ values decrease with the increase in % MWCNT. The sensor size did not influence the sensor response, while the % MWCNT affected the sensor behavior upon relative humidity (RH) increments. The 1% MWCNT/PVA sensor showed the best response, reaching a relative electrical resistance, ΔR/R_0_, of 509% at 99% RH. Comparable with other reported sensors, the produced MWCNT/PVA flexible sensor is simpler, greener and shows a good sensitivity to humidity, being easily incorporated in wearable monitoring applications, from sports to medical fields.

## 1. Introduction

Nowadays, there is a high demand for wearable applications with real-time feedback; from smart watches to insoles and even smart clothes, the possibilities are limitless. Physical information is obtained through the integration of sensors, e.g., temperature, humidity and heart rate, that can be used for medical diagnoses, to assess performance and even assess physical comfort. Flexible sensors are more suitable for these kinds of applications due to their higher mechanical resistance and conformable shape, allowing people free movement and higher comfort. Flexible humidity sensors represent a massive gap in the commercial field; thus, there is a need to develop simple and low-cost humidity sensors suitable for several wearable applications.

Researchers have already reported several materials suitable for sensor integration and humidity measurements: poly (ethylene glycol) (PEG)/gold nanoparticles [1]; UV-curable electrolytes inks [2]; polyaniline (PANI) ink [3]; cellulose acetate butyrate (CAB) [4]; phtalocyanine [5]; poly (3,4-ethylenedioxythiophene) (PEDOT)/7-(4-vinylbenzyloxyl)-4-methylcoumarin (VM) and maleic anhydride (MA) [6]; titanium dioxide (TiO_2_) [7]; paper [8]; graphene oxide (GO) [9,10,11,12]; graphene [13], poly (methacrylic acid methyl ester) (PMMA) [14]; carbon black [15]; poly (2-hydroxyethyl methacrylate) (pHEMA) [16]; polyvinyl alcohol (PVA) [17]; poly (styrene-block-butadienstyrene) nanofibers (SBS NFs) and alkalized MXenes/polydopamine (AMP) composite [18]; carbon nanotubes (CNT) ink [19]; and carbon nanotubes, either single wall (SWCNT) [20,21] or multiwall (MWCNT) [22,23,24]. Some authors have reported the need to functionalize carbon nanotubes to improve their dispersion in matrices and enhance sensor performance. Multiwall carbon nanotubes (MWCNTs) have been used for their outstanding electrical properties and high surface area, which can increase the sensor’s sensitivity and range. Used as the active layer of a humidity sensor, MWCNTs are commonly dispersed in a matrix, e.g., hydroxyethyl cellulose (HEC) [15,25], poly (ethyleneimine) (PEI) [26], poly (acrylic acid) (PAA) [27], poly(vinylpyrrolidone) (PVP) [28], polyimide (PI) [29,30], chitosan and polyamidoamine (PAMAM) dendrimer G3 (PM) [31] and polyvinyl alcohol (PVA) [32,33]. Some common techniques used to produce these flexible sensors are printing methods (e.g., inkjet printing, screen printing, gravure printing), coating methods (e.g., spin coating, spray coating), and casting. The active materials and production techniques, as well as their sensitivity towards the humidity of resistive-type humidity sensors, are summarized in Table 1. Despite the advances made in this field, several limitations need to be overcome, from the use of expensive materials, their complex production and non-reproducibility, the need for low-temperature and greener manufacturing processes, to sensor performance, e.g., sensitivity and range. From all the reports, the simple combination of MWCNTs with a water-soluble polymer, PVA, has shown promising results [32,33].

In this paper, a flexible MWCNT/PVA-based humidity sensor is proposed. The PVA was chosen for being cheap, having simple handling and being an eco-friendly polymer sensitive to humidity. MWCNTs were dispersed in the PVA solution to enhance the humidity sensor’s performance and stability by creating a conductive network within the PVA matrix, facilitating electron transport.

The effect of the sensor size and MWCNT percentage on the sensor’s electrical properties and response to the relative humidity levels was assessed. The sensing mechanisms that characterize sensor performance are discussed. The morphology of the MWCNT/PVA films with different percentages of MWCNT was observed by scanning electron microscopy (SEM). A simple, low-cost, more sustainable and flexible MWCNT/PVA-based humidity sensor with high sensitivity was obtained.

## 2. Materials and Methods

### 2.1. Materials

The interdigitated electrodes (IDTs) were screen printed with silver ink from Dycotec, Calne, UK (DM-SIP-2002). The polymeric substrate used was polyimide (PI), with a 75 µm thickness, from Addev Materials, Żychlin, Poland. Polyvinyl alcohol (PVA) (medium molecular weight) from Alfa Aesar (Ward Hill, MA, USA) and multiwall carbon nanotubes (MWCNT) from Nanocyl, Sambreville, Belgium (NC7000^TM^) were used as the humidity-sensing layer.

### 2.2. Sensor Fabrication

**Figure 1:**
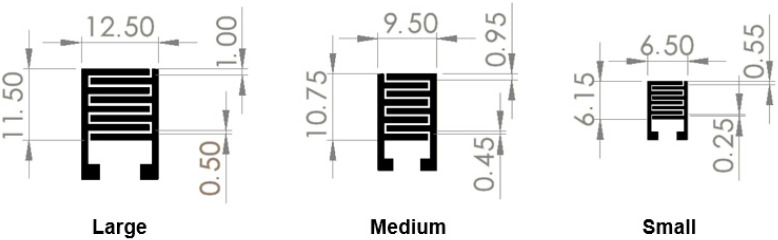
Schematics of the screen-printed interdigitated electrodes (IDTs) with different sizes: large, medium and small. Dimensions in millimeters (mm).

Interdigital electrodes (IDTs) were screen printed on top of the polyimide (PI) thin film using silver ink. Different IDT sizes were printed, maintaining the number of fingers (Figure 1).

**Figure 2:**
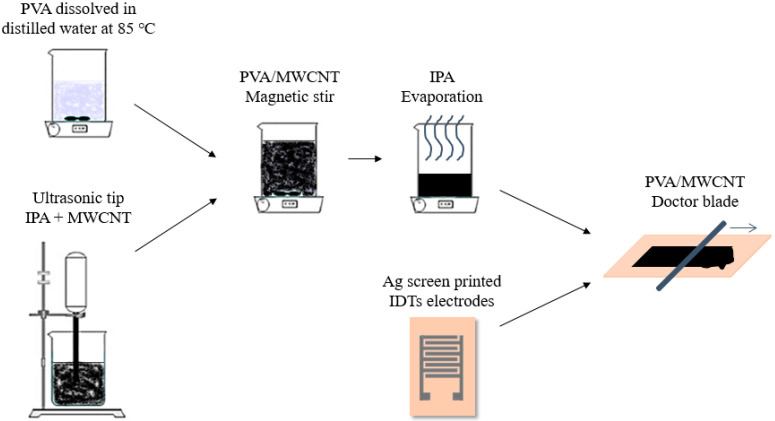
Schematics of the MWCNT/PVA-based humidity sensor production process.

The 4% PVA solutions were produced by dissolving PVA in distilled water at 85 °C. The MWCNTs were dispersed in isopropyl alcohol (IPA) with an ultrasonic tip for 30 min and then added to the respective 4% PVA solution. The mixtures were vigorously stirred until the IPA was totally evaporated. The MWCNT/PVA blends were cast on top of the screen-printed silver ITDs using a rectangular polyimide mask tape and cured for 2 h in a convection oven (Venticell 55) at 60 °C (Figure 2). A MWCNT/PVA mixture containing 0.5% MWCNTs was used to produce three different sized sensors: large, medium and small; these had a respective active sensing layer of 14 *×* 15 mm, 11 × 12 mm and 8 × 8 mm. Moreover, medium-sized MWCNT/PVA sensors were produced using different percentages of MWCNT: 1%, 0.5%, 0.4% and 0.25%.

### 2.3. Sensor Morphological Characterization

Scanning electron microscopy (SEM) was performed to assess the morphological characteristics of the MWCNT/PVA thin films with different percentages of MWCNT (1%, 0.5%, 0.4% and 0.25%).

### 2.4. Electrical Resistance Measurements

#### 2.4.1. Initial Electrical Resistance Measurements

To assess the influence of different sizes (large, medium and small) and percentages of MWCNT (1%, 0.5%, 0.4% and 0.25%) in the initial electrical properties of the MWCNT/PVA sensors, electrical resistance measurements were performed using a 2-point digital multimeter, Fluke 8846A, at room conditions.

#### 2.4.2. Electrical Resistance under Humidity Levels

The humidity measurements were performed inside a desiccator in a controlled environment. Saturated salt solutions were used to induce different relative humidity (RH) percentages, from 33% RH to 97% RH [37]; these values were confirmed by a hygrometer (Table 2). Once the humidity level stabilized after 24 h, the electrical resistance ($R_{\%RH}$) values of each sensor were measured. Those resistance ($R_{\%RH}$) values were used to calculate the relative electrical resistance ($∆R/R_{0}=\frac{\left(R_{\%RH}-R_{0}\right)}{R_{0}}\times 100\%$) to analyze the sensor’s behaviour as the RH increased. The R_0_ is the electrical resistance at a 35% relative humidity (RH) level.

## 3. Results and Discussion

### 3.1. Humidity Sensor Size Assessment

The effect of the sensor size was assessed for the 0.5% MWCNT samples with different sensor sizes: large, medium and small.

#### 3.1.1. Initial Electrical Resistance Measurements

**Figure 3:**
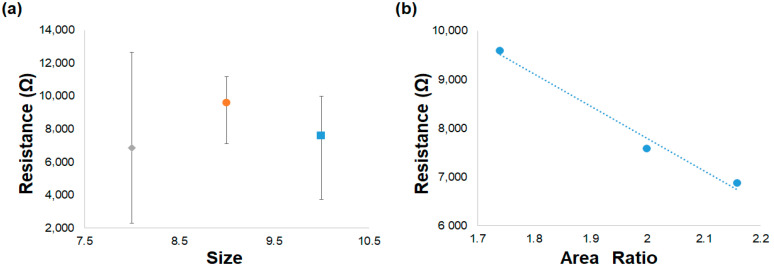
(**a**) Initial electrical resistance of the MWCNT/PVA sensors with different sizes: small (♦), medium (●) and large (■); (**b**) area ratio for the three sensor sizes.

The initial value of the electrical resistance was measured at room temperature for the 0.5% MWCNT samples with different sensor sizes: large, medium and small. The medium electrical resistance values and respective errors are presented in Figure 3a; the results showed some variability due to the MWCNT dispersion within PVA matrix. A close relationship between the printed sensor size and the electrical resistance behavior was not obtained. The area ratio between the active sensor layer (A_MWCNT/PVA_), which corresponds to the geometric area occupied by the sensor, and the silver interdigitated electrode (A_IDTs_) areas were calculated using Equation (1).

(1) $$Area\;Ratio=\frac{A_{MWCNT/PVA}}{A_{IDTs}}$$

This area ratio corresponds to the paths that the electrical current needs to travel inside the active layer, between the conductive lines of the IDT electrodes. In Table 3, the A_MWCNT/PVA_ and A_IDTs_ and their area ratio are specified according to sample size. Figure 3b presents the relationship between the initial electrical properties of the samples and their area ratio. The initial electrical resistance decreases linearly with the increase in the area ratio due to the increase in the MWCNT network present in the active layer material.

#### 3.1.2. Electrical Resistance under Different Humidity Levels

**Figure 4:**
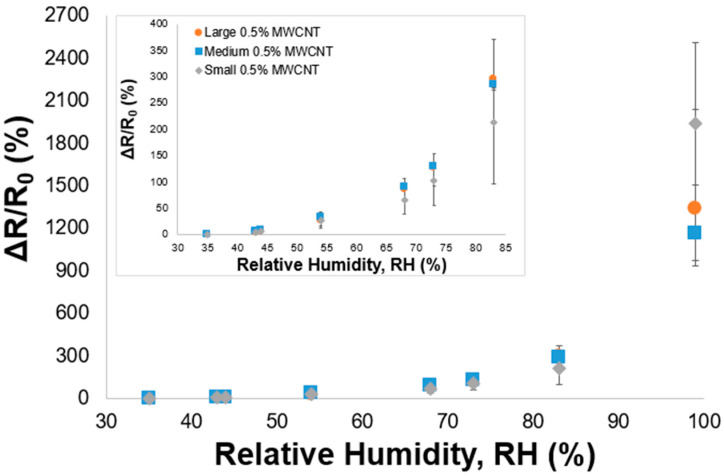
Relative electrical resistance variation, ΔR/R_0_, of the 0.5% MWCNT/PVA samples (three sizes: large—●, medium—■ and small—♦) vs. relative humidity (RH). Inset shows the amplified ΔR/R_0_ curves at lower RH% levels.

The 0.5% MWCNT/PVA sensors with different sizes were tested in several relatively humid environments. For each type, three samples were tested; the respective medium values and errors of the relative electrical resistance upon RH increments are presented in Figure 4.

The large, medium and small samples presented a similar behavior: there was an exponential increase in the relative electrical resistance upon an increase in the relative humidity, reaching a $∆R/R_{0}$ of 1938% at 99% RH. Regarding the reproducibility of each sample size, represented by the error bars, a slight variation that is more noticeable above 73% RH was observed, probably due to the non-homogeneous dispersion of MWCNTs within the PVA matrix.

### 3.2. MWCNT Percentage Assessment

The effect of the percentage of MWCNTs incorporated (1%, 0.5%, 0.4% and 0.25%) in the medium sensor was assessed.

#### 3.2.1. Morphological Characterization SEM

**Figure 5:**
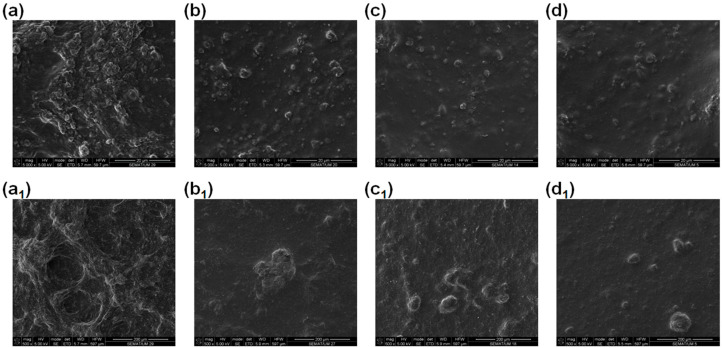
SEM characterization of the MWCNT/PVA with different MWCNT percentages: (**a**,**a_1_**) 1% MWCNT; (**b**,**b_1_**) 0.5% MWCNT; (**c**,**c_1_**) 0.4% MWCNT; (**d**,**d_1_**) 0.25% MWCNT, with 5000× and 500,000× magnification, respectively.

Scanning electron microscopy (SEM) was performed to assess the influence of the different percentages of MWCNTs on the morphological characteristics of the MWCNT/PVA thin films. In Figure 5, the increase in the number and size of the MWCNT agglomerates with the increase in the MWCNT percentage is noticeable. Moreover, compact mountain-like structures are observed when the MWCNT percentage reaches 1% and a smoother surface is observed in the samples with only 0.25% MWCNTs.

#### 3.2.2. Initial Electrical Measurements

**Figure 6:**
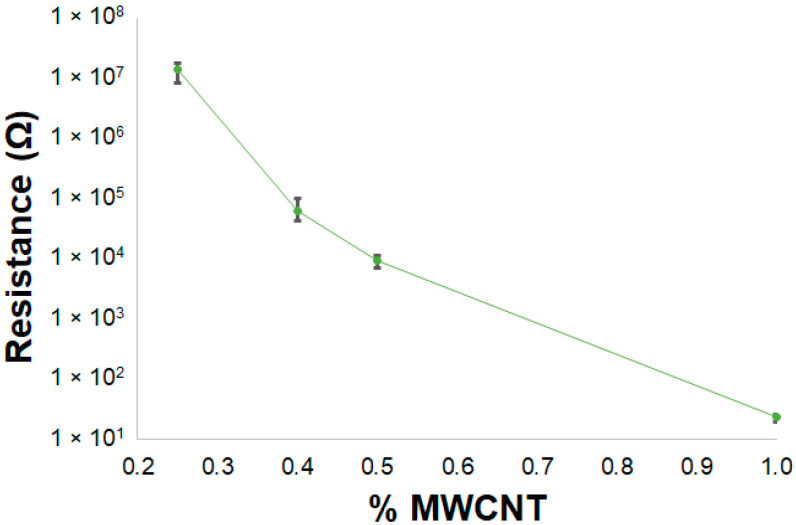
Variation in the initial electrical resistance of the MWCNT/PVA samples with % MWCNT.

The initial electrical resistance value of the MWCNT/PVA sensors with different percentages of MWCNT (1%, 0.5%, 0.4% and 0.25%) was measured at room temperature. Three samples were measured for each sensor; their medium electrical resistance values and respective errors are presented in Figure 6. The electrical resistance values decreased by 10^6^ orders of magnitude with an increase in the MWCNT percentage from 0.25% to 1%. At 1% MWCNT, there is the formation of a highly connected network of electrical paths that reduces the electrical resistance of the MWCNT/PVA composite. As can be seen by the small size of the error bars, the results presented low variability for each sensor type, which can indicate consistency in the sensor production and MWCNT dispersion within the PVA matrix.

#### 3.2.3. Electrical Measurements under Humidity

The MWCNT/PVA sensors with different percentages of MWCNT (1%, 0.5%, 0.4% and 0.25%) were tested regarding their response to different humidity levels; three sensor samples were tested for each MWCNT percentage. The response of a humidity sensor based on PVA and MWCNTs is the result of different effects; some are negligible, and others can dictate the sensor behavior. The main effects are as follows:

- PVA Matrix Swelling: As humidity increases, the hydrophilic PVA matrix absorbs water molecules and swells. This swelling increases the separation between the MWCNTs dispersed in the PVA, reducing the number of conductive paths between them. As a result, the overall electrical resistance of the sensor increases due to the reduced connectivity within the MWCNT network [33].
- MWCNT Water Absorption: The MWCNTs absorb water molecules, which leads to an increase in their electrical resistance. This occurs because the adsorbed water molecules alter the charge carrier density and mobility within the MWCNT network, which interferes with the electrical conductivity of the MWCNTs [22].
- Decreased PVA Resistance: On the other hand, the electrical resistance of the PVA matrix decreases non-linearly with increasing humidity. This is because the presence of absorbed water molecules enhances the ionic conductivity within the PVA matrix [17,32].

**Figure 7:**
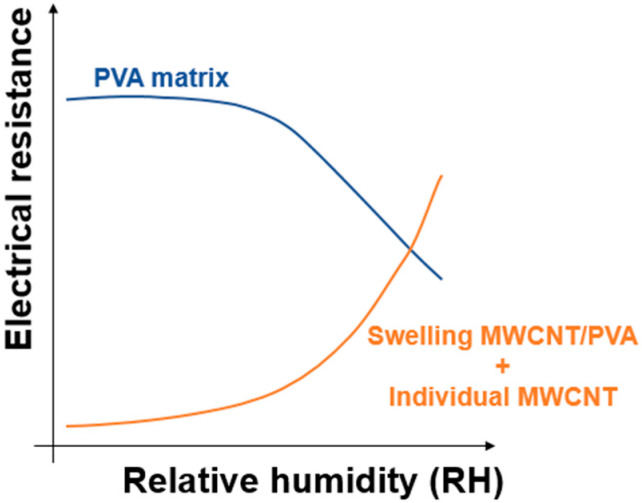
Electrical resistance behavior of PVA matrix, individual MWCNTs and the MWCNT/PVA upon RH increment.

The overall sensor response is governed by the relative magnitude of these effects, which vary depending on the humidity level and the concentration of MWCNTs in the composite. At a higher humidity, the swelling of the PVA matrix plays a more significant role in increasing the resistance, whereas at a lower humidity, the water absorption by MWCNTs is more prominent. The variation in electrical resistance with respect to % RH is, therefore, a balance of these competing phenomena, as also discussed in the following sections and illustrated in Figure 7.

**Figure 8:**
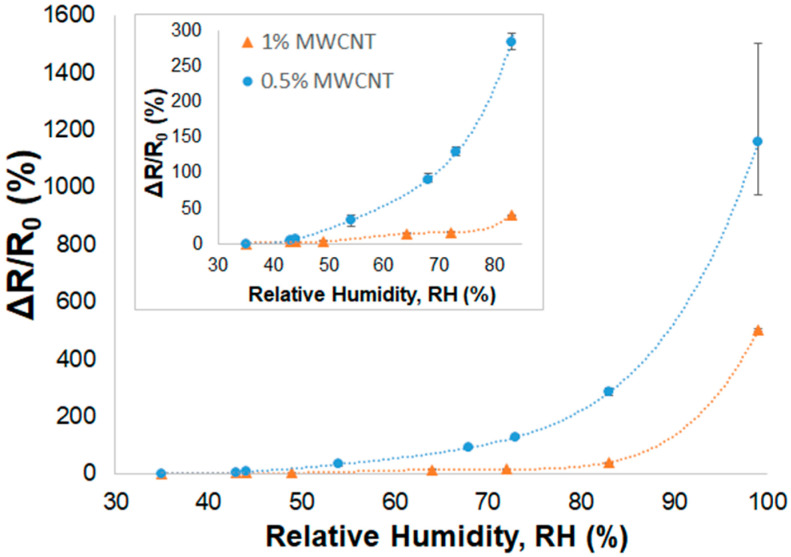
Relative electrical resistance, ΔR/R_0_, of MWCNT/PVA samples: 1% MWCNT (▲) and 0.5% MWCNT (●) vs. relative humidity (RH). Inset shows the amplified ΔR/R_0_ curves at lower RH% levels.

**Figure 9:**
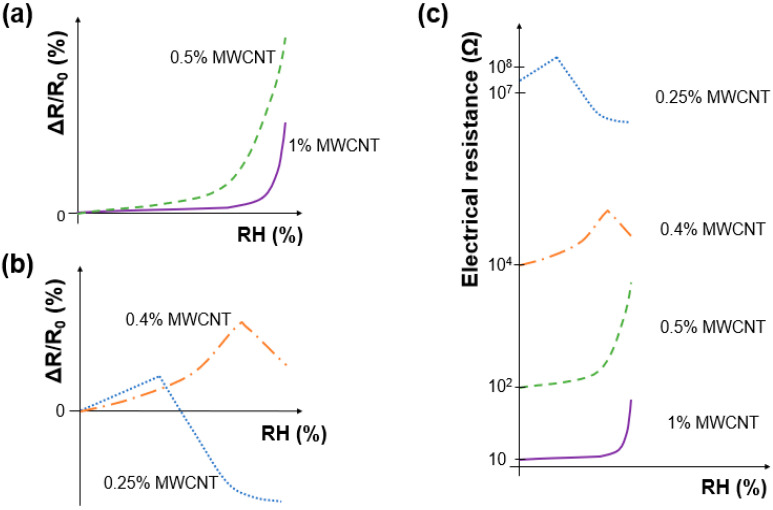
Influence of % MWCNT on the MWCNT/PVA sensor’s response upon an increase in RH: (**a**) relative electrical resistance, ΔR/R_0_, of high % MWCNT sensor; (**b**) relative electrical resistance, ΔR/R_0_, of low % MWCNT sensor and (**c**) comparison of electrical resistance behavior of MWCNT/PVA sensors.

The relative electrical resistances ($∆R/R_{0}$) upon relative humidity increments for three samples of 1% and 0.5% MWCNT/PVA sensors (▲ and ●, respectively) are plotted in Figure 8. Both types of sensors showed an almost exponential increase in the $∆R/R_{0}$ values with RH (%) increase, showing a good sensitivity to humidity even at lower relative humidity levels. The 1% MWCNT/PVA samples present very low variability, while in the 0.5% MWCNT/PVA, the small effect of the non-homogeneous MWCNT dispersion can be observed, as this influences its humidity response at high 99% RH. In both cases, the sensor resistive response is due to the MWCNT separation caused by PVA swelling, which is caused by the increase in RH that causes an increase in the sensor’s overall electrical resistance [33]. The 0.5% MWCNT/PVA sensor has a higher sensitivity (1500%) to humidity compared to the sensor with 1% MWCNT (509%), since the lower percentage of MWCNT facilitates the disruption of the conduction paths due to PVA swelling and induces a higher variation in $∆R/R_{0}$ for the same relative humidity level. As shown more clearly in Figure 9a, the sensor’s exponential behavior starts to be more pronounced at lower RH percentages in the 0.5% MWCNT/PVA (between 60 and 70% RH) compared to the 1% MWCNT/PVA (above 80% RH).

**Figure 10:**
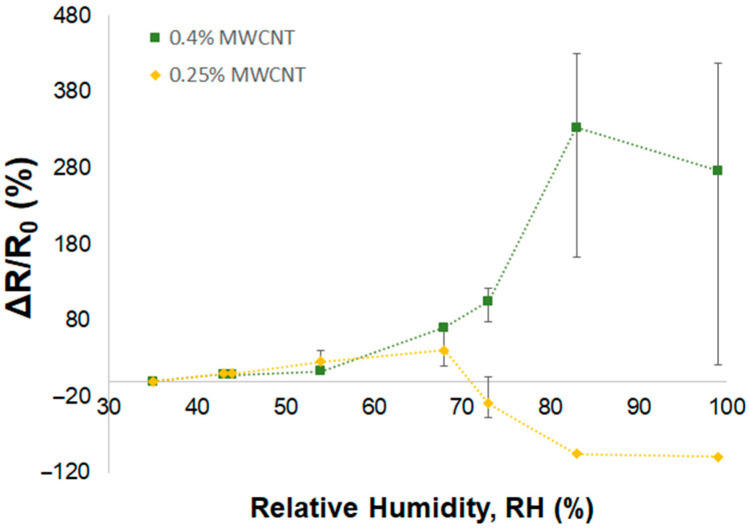
Relative electrical resistance, ΔR/R_0_, of MWCNT/PVA samples: 0.4% MWCNT (■) and 0.25% MWCNT (♦) vs. relative humidity (RH).

The relative electrical resistances ($∆R/R_{0}$) caused by an increase in the relative humidity for the 0.4% and 0.25% MWCNT/PVA sensors are plotted in Figure 10, respectively. Both sensors showed an increase in their $∆R/R_{0}$ values with the increase in the relative humidity levels until reaching a certain RH (%) value, approximately 83% RH and 67% RH for the 0.4% and 0.25% MWCNT/PVA sensors, respectively, after which the relative resistance started to decrease. At low humidity levels, the effect of PVA swelling on the MWCNT conductive path was dominant and the sensor’s overall resistance increased with the increase in humidity. When a specific humidity level was reached, another humidity-sensing mechanism becomes dominant, and the sensor’s overall resistance decreases due to the decrease in PVA resistance upon % RH increment. The lower the MWCNT percentage present in the sensor, the lower the specific turning point of the relative humidity level (Figure 9b), and the more dominant the effect of PVA resistance decreasing with an increase in RH. The results showed high variability, being above 73% RH and between 43 and 83% RH for the sensors with a lower percentage of MWCNT, at 0.4 and 0.25%, respectively. The variability in the sensor production due to the non-homogeneous dispersion and structure of MWCNTs is more evident in the sensors with a low percentage of MWCNTs.

The electrical conduction mechanisms of the humidity sensors are dependent upon the % of the MWCNT incorporated. This is depicted in Figure 9c. At a low % of MWCNT (in this case, 0.25–0.4%), the initial electrical resistance values are high and the effect that has the tendency to become dominant (from a particular RH point) is the PVA resistance decreasing with an increase in RH.

For a high % of MWCNT incorporated in the PVA (in this case 0.5–1%), the initial electrical resistance values are smaller and the effect of PVA swelling, which affects the MWCNT network and therefore increases resistance as the RH increases, is dominant.

Despite the 0.5% MWCNT/PVA humidity sensor showing the highest sensitivity, the 1% MWCNT/PVA presented the best outcome, combining the lowest variability and a good sensitivity (high ΔR/R_0_ (%) of 41% at 83% RH, reaching 509% at 99% RH). A comparison between the present results and other reports [32,33] is presented in Table 4. A functionalized MWCNT/PVA nanocomposite-based humidity sensor [32] was produced with 5% MWCNT, and this had a high initial electrical resistance of 5 × 10^11^ Ω, presenting a ΔR/R_0_ (%) of −100% at 94% RH. This negative dependence of the electrical response upon RH increment is due to the dominant humidity sensing mechanism of PVA. At these high electrical resistance values, the PVA resistance decreases with an increase in RH, covering the effect of the MWCNT network. In contrast, a functionalized MWCNT-COOH/PVA-based sensor [33] was produced with 5% MWCNT, with an initial electrical resistance of 6 × 10^4^ Ω and a ΔR/R_0_ (%) of 488% at 100% RH. The sensor presented an exponential increase in ΔR/R_0_ (%) upon RH increment due to the decrease in MWCNT conductive paths upon PVA swelling with RH increment. This MWCNT-COOH/PVA humidity sensor [33] and the 1% MWCNT/PVA of this work presented a similar response to humidity; however, the developed 1% MWCNT/PVA humidity sensor reached a sensitivity of 509% at 99% RH, without MWCNT functionalization and using a lower percentage of MWCNT. Moreover, it presents a lower initial electrical resistance (R_0_), which facilitates its integration into humidity-sensing applications.

## 4. Conclusions

A humidity sensor based on MWCNTs dispersed in a PVA matrix was produced. The effect of the sensor size was assessed, and a correlation between the area ratio and the initial electrical resistance of the sensor was obtained; however, there was no influence on its sensing performance. Moreover, the effect of the MWCNT percentage on the morphological and electrical properties of the sensor was studied. The SEM analysis showed noticeable morphological differences between the sensors with different percentages of MWCNT; e.g., compact MWCNT structures are observed in the sensor samples with 1% MWCNT compared with the smoother surface of the samples with 0.25% MWCNT. However, all the produced sensors with different MWCNT percentages showed high sensitivity to humidity, and their sensing response depended highly on the MWCNT percentage and relative humidity levels. Two opposite effects in the humidity response of MWCNT/PVA sensors need to be considered: (a) the increase in the overall sensor electrical resistance with increased humidity due to the interference of PVA swelling in the MWCNT conductive paths, and (b) the decrease in the PVA electrical resistance with increased humidity. In the sensors with a higher percentage of MWCNT (1% and 0.5%), the swelling effect was dominant, while in the sensors with a lower percentage of MWCNT (0.4% and 0.25%), the increase in PVA resistance with humidity became dominant from a specific humidity level. Some variability in the results was observed, especially for the sensors with a lower MWCNT percentage due to the non-homogeneity of sensor production. The MWCNT/PVA sensor that showed the best combination of a humidity sensing response and low production variability was the 1% MWCNT/PVA. As a result, a highly sensitive MWCNT/PVA-based humidity sensor was obtained through a simple production process, without MWCNT functionalization; this sensor is suitable for wearable humidity-sensing applications.

## Figures and Tables

**Table 1:** Resistive-type humidity sensors, production methods and humidity response.

Materials	Production Method	Linearity	Sensitivity	Reference
PEG/Gold NP	Inkjet printing	Non-Linear	10^5^ variation from 1.8 to 95 RH%	[1]
CNT ink	Gravure printing	Linear	0.1%/% RH	[19]
UV-curable electrolytes inks	Inkjet printing	Linear	10^3^ variation from 30 to 90 RH%	[2]
PANI ink	Inkjet printing	Non-Linear	308 Ω/RH%	[3]
PEDOT:PVMA	Inkjet printing	Linear	−71.85%~−98.46% from 11~98%	[6]
TiO_2_	Screen printing	Linear	4 orders of magnitude change from 24 to 90% RH	[7]
PEDOT: PSS; Methyl Red; GO	Spin coating	Non-Linear	180 kΩ/% RH	[9]
GO	Drop casting	Non-Linear	811 Ω/% RH	[10]
Functionalized-MWCNT/HEC	Gravure printing	Linear	0.048/% RH	[25]
HEC/MWCNT	Casting	Linear	0.10069	[15]
HEC/CBs	Casting	Linear	0.10692	
pHEMA	Gravure press	Non-Linear	172% at 80% RH	[16]
PEI/oxidized MWCNT	Layer-by-layer(LbL) assembly	Non-Linear	47 Ω/% RH	[26]
Untreated and chemically treated MWCNT	Spraying	Linear	120% resistance increase (sensitivity 1.34)	[22]
Dispersed MWCNT	Dielectrophoresis	Linear	0.5%/% RH	[23]
MWCNT/PAA	Casting	Linear	0.069/% RH	[27]
SWCNT/PVA	Wet spinning	Non-Linear	24 times higher from 60 to 100 RH%	[20]
SWCNT/epoxy	Vacuum filtration	Linear	0.03/% RH	[21]
Plasma-treated-MWCNT/PI	Spin coating	Linear	0.0047/% RH	[29]
Functionalized-MWCNT/PI	Spin coating	Linear	60 Ω/% RH	[34]
PI/MWCNT	In situ polymerization	Linear	0.00146/% RH	[30]
MWCNT/PVP	Spin coating	Non-Linear	4000%	[28]
MWCNT/CNF	Near-field electrohydrodynamic direct writing (NFEDW)	Non-Linear	61.5% (ΔR/R_0_) at 95 RH%	[24]
PVA	Spin coating	Non-Linear	10^5^ variation from 35 to 94 RH%	[17]
Functionalized-MWCNT/PVA	Casting	Non-Linear	10^5^ variation from 11 to 94% RH.	[32]
CNT-COOH/PVA	Casting	Non-Linear	Switch 80–90% RH 32.3 at 100% RH	[33]
CNT ink/PVP; CNT ink/PVA and CNT ink/gelatin	Spin coating	Non-Linear	86, 48, and 31 Ω/% RH	[35]
CNT-OH/crosslinked PVA	Spin coating	Linear	99.97% from 40–86% RH.	[36]

**Table 2:** Salts used to induce different humidity percentages in the desiccator environment.

Salt	RH (%)	
	Theoretical [37]	Hygrometer
Magnesium chloride (MgCl_2_)	33	35
Potassium carbonate (K_2_CO_3_)	44	44
Sodium chloride (NaCl)	76	73
Potassium chloride (KCl)	86	83
Potassium sulphate (K_2_SO_4_)	97	99

**Table 3:** Relationship between the IDTs and active sensor layer (MWCNT/PVA) area.

Size	A_IDTs_ (mm^2^)	A_MWCNT/PVA_ (mm^2^)	Area Ratio
Small	29.65	64	2.16
Medium	75.72	132	1.74
Large	105	210	2

**Table 4:** MWCNT/PVA-based humidity sensors.

Material	MWCNT %	Humidity Level (%)	Initial Resistance, R_0_ (Ω)	ΔR/R_0_ (%)	Dependence	Reference
Functionalized MWCNT/PVA nanocomposite	5	11–50	5 × 10^11^	−90	Negative	[32]
		50–94	5 × 10^10^	−100		
MWCNT-COOH/PVA	5	11–75	6 × 10^4^	41	Positive	[33]
		75–100	8.5 × 10^4^	488		
CNT ink/PVA	2	11–96		110	Positive	[35]
CNT-OH/crosslinked PVA	1	40–84	10^8^	−99.97	Negative	[36]
MWCNT/PVA	1	35–83	24	41	Positive	This work
		83–99	33	509		

## Data Availability

Data are contained within the article.

## References

[1] Su C.H., Chiu H.L., Chen Y.C., Yesilmen M., Schulz F., Ketelsen B., Vossmeyer T., Liao Y.C. (2019). Highly Responsive PEG/Gold Nanoparticle Thin-Film Humidity Sensor via Inkjet Printing Technology. Langmuir.

[2] Cho N.B., Lim T.H., Jeon Y.M., Gong M.S. (2008). Inkjet Printing of Polymeric Resistance Humidity Sensor Using UV-Curable Electrolyte Inks. Macromol. Res..

[3] Kulkarni M.V., Apte S.K., Naik S.D., Ambekar J.D., Kale B.B. (2013). Ink-Jet Printed Conducting Polyaniline Based Flexible Humidity Sensor. Sens. Actuators B Chem..

[4] Kinkeldei T., Mattana G., Leuenberger D., Ataman C., Lopez F.M., Quintero A.V., Briand D., Nisato G., de Rooij N.F., Tröster G. (2012). Feasibility of Printing Woven Humidity and Temperature Sensors for the Integration into Electronic Textiles. Adv. Sci. Technol..

[5] Soukup R., Hamáček A., Řeboun J. Advanced Screen Printing for the Fabrication of Organic Humidity Sensors. Proceedings of the 2012 4th Electronic System-Integration Technology Conference, ESTC 2012.

[6] Yuan Y., Zhang Y., Liu R., Liu J., Li Z., Liu X. (2016). Humidity Sensor Fabricated by Inkjet-Printing Photosensitive Conductive Inks PEDOT:PVMA on a Paper Substrate. RSC Adv..

[7] Dubourg G., Katona J., Rodović M., Savić S., Kitic G., Niarchos G., Jancović N., Crnojević-Bengin V. (2017). Flexible and Highly Sensitive Humidity Sensors Using Screen-Printed TiO2 Nanoparticles as Sensitive Layer. J. Phys. Conf. Ser..

[8] Gaspar C., Olkkonen J., Passoja S., Smolander M. (2017). Paper as Active Layer in Inkjet-Printed Capacitive Humidity Sensors. Sensors.

[9] Hassan G., Sajid M., Choi C. (2019). Highly Sensitive and Full Range Detectable Humidity Sensor Using PEDOT:PSS, Methyl Red and Graphene Oxide Materials. Sci. Rep..

[10] Descent P., Izquierdo R., Fayomi C. Printing of Temperature and Humidity Sensors on Flexible Substrates for Biomedical Applications. Proceedings of the IEEE International Symposium on Circuits and Systems 2018.

[11] He P., Brent J.R., Ding H., Yang J., Lewis D.J., O’Brien P., Derby B. (2018). Fully Printed High Performance Humidity Sensors Based on Two-Dimensional Materials. Nanoscale.

[12] Chen H., Han K., Li Y. (2023). Laser-Direct-Writing Assisted Preparation of Flexible Humidity Sensors Based on Semi-Interpenetrating Polymer Network for Applications in Non-Contact Human-Machine Interaction. Sens. Actuators B Chem..

[13] Wang H., Tang C., Xu J. (2023). A Highly Sensitive Flexible Humidity Sensor Based on Conductive Tape and a Carboxymethyl Cellulose@graphene Composite. RSC Adv..

[14] Reddy A.S.G., Narakathu B.B., Atashbar M.Z., Rebros M., Rebrosova E., Bazuin B.J., Joyce M.K., Fleming P.D., Pekarovicova A. (2011). Printed Capacitive Based Humidity Sensors on Flexible Substrates. Sens. Lett..

[15] Ma X., Ning H., Hu N., Liu Y., Zhang J., Xu C., Wu L. (2015). Highly Sensitive Humidity Sensors Made from Composites of HEC Filled by Carbon Nanofillers. Mater. Technol..

[16] Reddy A.S.G., Narakathu B.B., Atashbar M.Z., Rebros M., Rebrosova E., Joyce M.K. (2011). Fully Printed Flexible Humidity Sensor. Procedia Eng..

[17] Martadi S., Sulthoni M.A., Wiranto G., Surawijaya A., Herminda I.D.P. Design and Fabrication of PVA-Based Relative Humidity Sensors Using Thick Film Technology. Proceedings of the 2019 International Symposium on Electronics and Smart Devices, ISESD 2019.

[18] Li T., Zhao T., Zhang H., Yuan L., Cheng C., Dai J., Xue L., Zhou J., Liu H., Yin L. (2024). A Skin-Conformal and Breathable Humidity Sensor for Emotional Mode Recognition and Non-Contact Human-Machine Interface. npj Flex. Electron..

[19] Turkani V.S., Narakathu B.B., Maddipatla D., Bazuin B.J., Atashbar M.Z. A Fully Printed CNT Based Humidity Sensor on Flexible PET Substrate. Proceedings of the 17th International Meeting on Chemical Sensors—IMCS 2018.

[20] Zhou G., Byun J.H., Oh Y., Jung B.M., Cha H.J., Seong D.G., Um M.K., Hyun S., Chou T.W. (2017). Highly Sensitive Wearable Textile-Based Humidity Sensor Made of High-Strength, Single-Walled Carbon Nanotube/Poly(Vinyl Alcohol) Filaments. ACS Appl. Mater. Interfaces.

[21] Arunachalam S., Gupta A.A., Izquierdo R., Nabki F. (2018). Suspended Carbon Nanotubes for Humidity Sensing. Sensors.

[22] Cao C.L., Hu C.G., Fang L., Wang S.X., Tian Y.S., Pan C.Y. (2011). Humidity Sensor Based on Multi-Walled Carbon Nanotube Thin Films. J. Nanomater..

[23] Liu L., Ye X., Wu K., Han R., Zhou Z., Cui T. (2009). Humidity Sensitivity of Multi-Walled Carbon Nanotube Networks Deposited by Dielectrophoresis. Sensors.

[24] Pan T., Yu Z., Huang F., Yao H., Hu G., Tang C., Gu J. (2023). Flexible Humidity Sensor with High Sensitivity and Durability for Respiratory Monitoring Using Near-Field Electrohydrodynamic Direct-Writing Method. ACS Appl. Mater. Interfaces.

[25] Turkani V.S., Maddipatla D., Narakathu B.B., Saeed T.S., Obare S.O., Bazuin B.J., Atashbar M.Z. (2019). A Highly Sensitive Printed Humidity Sensor Based on a Functionalized MWCNT/HEC Composite for Flexible Electronics Application. Nanoscale Adv..

[26] Yu H., Cao T., Zhou L., Gu E., Yu D., Jiang D. (2006). Layer-by-Layer Assembly and Humidity Sensitive Behavior of Poly(Ethyleneimine)/Multiwall Carbon Nanotube Composite Films. Sens. Actuators B Chem..

[27] Lee J., Cho D., Jeong Y. (2013). A Resistive-Type Sensor Based on Flexible Multi-Walled Carbon Nanotubes and Polyacrylic Acid Composite Films. Solid State Electron..

[28] Pan X., Xue Q., Zhang J., Guo Q., Jin Y., Lu W., Li X., Ling C. (2016). Effective Enhancement of Humidity Sensing Characteristics of Novel Thermally Treated MWCNTs/Polyvinylpyrrolidone Film Caused by Interfacial Effect. Adv. Mater. Interfaces.

[29] Yoo K.P., Lim L.T., Min N.K., Lee M.J., Lee C.J., Park C.W. (2010). Novel Resistive-Type Humidity Sensor Based on Multiwall Carbon Nanotube/Polyimide Composite Films. Sens. Actuators B Chem..

[30] Tang Q.Y., Chan Y.C., Zhang K. (2011). Fast Response Resistive Humidity Sensitivity of Polyimide/Multiwall Carbon Nanotube Composite Films. Sens. Actuators B Chem..

[31] Kim H.S., Kang J.H., Hwang J.Y., Shin U.S. (2022). Wearable CNTs—Based Humidity Sensors with High Sensitivity and Flexibility for Real—Time Multiple Respiratory Monitoring. Nano Converg..

[32] Manohara S.R., Samal S.S., Rudreshappa G.E. (2017). Humidity Sensing Properties of Multiwalled Carbon Nanotube/Polyvinyl Alcohol Nanocomposite Films. Nanosci. Nanotechnol.-Asia.

[33] Fei T., Jiang K., Jiang F., Mu R., Zhang T. (2014). Humidity Switching Properties of Sensors Based on Multiwalled Carbon Nanotubes/Polyvinyl Alcohol Composite Films. J. Appl. Polym. Sci..

[34] Myung J.L., Cheol-Jin L., Singh V.R., Kum-Pyo Y., Nam-Ki M. Humidity Sensing Characteristics of Plasma Functionalized Multiwall Carbon Nanotube-Polyimide Composite Films. Proceedings of the IEEE Sensors.

[35] Ding S., Yin T., Zhang S., Yang D., Zhou H., Guo S., Li Q., Wang Y., Yang Y., Peng B. (2023). Fast-Speed, Highly Sensitive, Flexible Humidity Sensors Based on a Printable Composite of Carbon Nanotubes and Hydrophilic Polymers. Langmuir.

[36] Ni Q., Zhang D., Shan G., Zheng Q., Du M. (2024). Preparation of PVA/CNTs Film with High Stability and Humidity Sensitivity Based on Multiple Process. Sens. Actuators A Phys..

[37] Greenspan L. (1977). Humidity Fixed Points of Binary Saturated Aqueous Solutions. J. Res. Natl. Bur. Stand. Sect. A Phys. Chem..

